# Loss of β-catenin in adrenocortical cancer cells causes growth inhibition and reversal of epithelial-to-mesenchymal transition

**DOI:** 10.18632/oncotarget.3222

**Published:** 2015-03-18

**Authors:** Aude Salomon, Michelle Keramidas, Cécile Maisin, Michaël Thomas

**Affiliations:** ^1^ Institut National de la Santé et de la Recherche Médicale, Unité 1036, Grenoble, France; ^2^ Commissariat à l'Energie Atomique, Institute of Life Sciences Research and Technologies, Biology of Cancer and Infection, Grenoble, France; ^3^ Université Grenoble Alpes, Unité Mixte de Recherche-S1036, Grenoble, France

**Keywords:** adrenocortical carcinoma, β-catenin, shRNA, epithelial-mesenchymal transition

## Abstract

Adrenal carcinoma (ACC) is a rare neoplasm with a poor outcome. Aberrant expression of β-catenin has been found in approximatively 30% of ACC. We herein studied its effects on the growth of the human ACC cell line H295R. The cells were infected with short hairpin RNA (shRNA)-mediated silencing β-catenin. Two shRNAs used induced down-regulation of β-catenin protein levels. The expression of these shRNAs decreased cell growth and increased H295R cells in S and G_2_/M phases. This cytostatic effect is due to a decrease of phosphorylated MAPK and to an up-regulation expression of the cyclin-dependent kinase inhibitors p57^KIP2^, p21^CIP^ and p27^KIP1^. In addition, the knockdown of β-catenin decreased phosphorylated Akt and increased apoptosis. Finally, loss of β-catenin was sufficient to induce the reversal of the epithelial-to-mesenchymal transition. We then transplanted these genetically modified H295R cells in Scid mice. Tumor growth suppression was achieved by the two shRNAs showing *in vitro* efficacy. Proliferation was not reduced in silenced tumors. In contrast, p57, p27 and p21 proteins were found expressed at high levels in silenced tumors along with an increase in apoptotic cells. These findings indicate that β-catenin loss in H295R cells inhibits tumor growth by inducing transcriptional and functional changes.

## INTRODUCTION

Human adrenocortical cancer (ACC) is one of the most aggressive malignant tumors with a generally poor prognosis related to tumor metastasis [1]. Despite significant advances to determine dysregulated gene expression associated with ACC by genome-wide gene expression profiling analysis, little of that considerable amount of data has been translated into the clinics and currently, surgical resection remains the mainstay of the curative modality for ACC patients [1]. Advancement in identifying molecular markers of ACCs has been made possible either by the study of hereditary tumor syndromes associated with adrenal neoplasms [2] or by the analysis of these tumors for gene alterations involved in the pathogenesis of other sporadic cancers [2].

Wnt signaling pathway plays an important role in differentiation, morphogenesis, and tumorigenesis [3]. One of the key mediators of this pathway is β-catenin, which plays a pivotal role in Wnt signal transduction in addition to its function as a cell-cell adhesion component. In normal cells, the cytoplasmic β–catenin is kept at low levels since the protein is recruited into a complex, including glycogen synthase kinase-3β, adenomatous polyposis coli protein, and axin, that leads to its phosphorylation and its subsequent degradation by ubiquitin-proteasome pathway [3]. Studies of adrenal tumors have demonstrated that accumulation of β–catenin in cytoplasm and nucleus is due to β–catenin gene (*CTNNB1*) mutations and represents the most frequent molecular event, with a prevalence of about 20% in both adenomas and ACCs [4, 5]. Such gene mutations affect predominantly phosphorylation sites yielding accumulation of free β-catenin which then interacts with the transcription factors, Tcf/Lef, and activates downstream target genes which are mainly involved in the regulation of cell fate and proliferation [6].

Poor therapeutic benefits of the available chemotherapy, and concerns about the safety and effectiveness of novel drugs trigger the development of new alternative methods to treat this deadly disease. In recent years, RNA interference (RNAi) has emerged as a powerful method of gene therapy, and has been widely used for silencing malignant cellular and viral genes [7, 8]. Advancement in RNAi is evident from the successful human trials with RNAi targeting VEGF and kinesin spindle protein in liver cancer patients [9].

In this report, we used RNA interference by stable expressing β-catenin small hairpin RNAs (β-catenin shRNAs) to analyze the roles of β-catenin in the malignant behaviors of human adrenal cancer cell line, H295R. We found a decrease in nuclear and cytoplasmic immunoreactivity of β-catenin in cells which led to reduced growth *in vitro*, mainly due to CDK inhibitors increase and p-MAPK decrease. On the other hand, knocking down of β-catenin expression in H295R cells reversed the epithelial-to-mesenchymal transition (EMT) through the up-regulation of the epithelial marker E-cadherin and the concomitantly decrease of mesenchymal markers vimentin and N-cadherin. In addition, silencing β-catenin reduced the migration and invasive potentials of the H295R cells *in vitro*. Finally the transplantation of these cells resulted in tumor formation of reduced size. Our data demonstrated that β-catenin expression is positively associated with growth and aggressiveness of human adrenal cancer cells and its suppression might be a potential therapeutic strategy for ACC.

## RESULTS

### Characterization of *in vitro* phenotype resulting from expression of β-catenin shRNA

**Figure 1:**
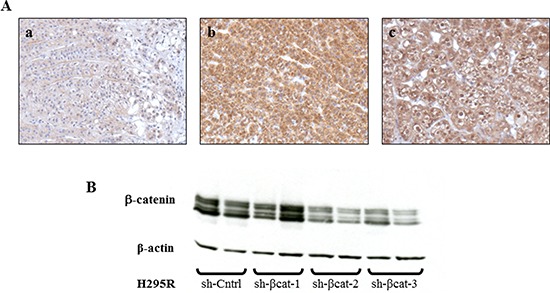
β-catenin expression in adrenocortical tissues and specific silencing of β-catenin mediated by stable expressing β-catenin shRNA **(A)** (a) Normal adrenal gland shows a specific membrane staining for β-catenin, magnification x200. (b) Adrenocortical carcinoma shows a strong cytoplasmic and nucleus β-catenin expression, magnification x200. (c) Tumor formed from H295R cells transplantation, removed at 28 days and stained for β-catenin, showing intense cytoplasmic and nucleus expression, magnification x200. **(B)** western blot analysis of H295R cell lysates shows a decreased expression of β-catenin with sh-βcat-2 and -3. β-Actin expression is used as a loading control.

A hallmark of active β-catenin signaling in the tumor is the accumulation of β-catenin both in the cytoplasm and nucleus irrespective of the mutational status of *CTNNB1*. Firstly, we examined the cellular localization of β-catenin in a normal human adrenal gland, a human adrenal carcinoma and xenografted tumors formed by transplantation of H295R cells beneath the kidney capsule of Scid mice. Whereas the β-catenin staining is essentially membranous in normal adrenal gland (Figure 1A(a)), β-catenin accumulates in the cytoplasm and nucleus of the adrenocortical carcinoma and the H295R tumor (Figure 1A(b, c)). To resolve the role of β–catenin constitutive activation in the pathogenesis of adrenocortical carcinoma, we used three independent β–catenin-targeting shRNA lentiviruses (sh-βcat-1,-2 and -3) in human cell line H295R. The efficiency of shRNA-mediated β-catenin knockdown was confirmed by western blot analysis for two out of three constructs (sh-βcat-2 and sh-βcat-3) resulting in > 80% reduction of β-catenin protein levels (Figure 1B). Therefore, the two sequences with the most effective silencing effect targeting β–catenin were chosen for knockdown expression of β–catenin and selected for further testing.

**Figure 2:**
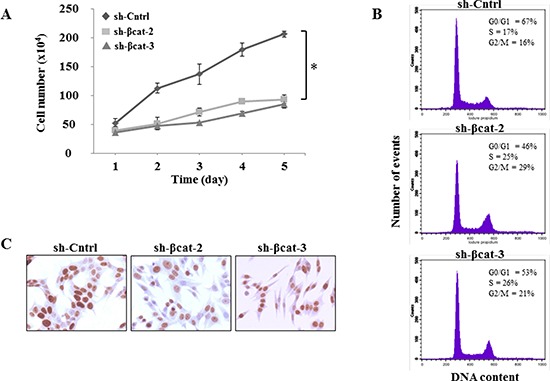
Characterization of *in vitro* phenotypes resulting from the expression of β-catenin shRNA **(A)** cells were seeded in triplicate, and counted 1, 2, 3, 4 and 5 days later by trypan blue exclusion *P<0.01. **(B)** effects of knockdown of β-catenin on cell cycle progression. Flow cytometry histograms show DNA content in H295R cells. **(C)** H295R cells expressing either sh-Cntrl, sh-βcat-2 or sh-βcat-3 were labeled with a monoclonal antibody against the nuclear proliferation marker Ki-67, as described in Materials and Methods section.

To address the biological relevance of the loss of β-catenin function, we monitored the effect of shRNA on the growth of H295R cells by counting cells every day for 5 days. The results of the cell count suggested that the growth in sh-Cntrl group was gradually increased in a time-dependent manner, but remained virtually unchanged in cells transfected with sh-β-catenin (Figure 2A). Statistical analysis indicated that the shRNA against β-catenin significantly inhibited the growth of H295R cells compared with the negative control (*p* < 0.01). To investigate the mechanism of growth inhibition of H295R cells by β-catenin shRNA, cell cycle analysis was performed using flow cytometry to determine the number of cells in different phases of the cell cycle (Figure 2B). In comparison to the control sh transfected cells, both β-catenin-shRNA transfections resulted in a decrease of sh-βcat-2 and -3 cells in G_0_/G_1_ phase (67%, 46% and 53%, respectively) with a concomitant increase of cells in S (17%, 25% and 26%, respectively) and G_2_/M (16%, 29% and 21%, respectively) phases (Figure 2B). These results indicate that treatment with shRNA directed against β-catenin induced arrest in S and G_2_/M phases in H295R cells. Moreover, the proliferation rate of the various cell populations was assessed by staining for the Ki-67 proliferation-associated protein. Although we did not make precise measurements of the proliferation indices, it was evident that the proliferation rate was very high and did not differ between sh-Cntrl and, sh-βcat-2 and -3 cells (Figure 2C). That also excluded a possible general cytostatic and cytotoxic effects caused by the extinction of β-catenin expression in H295R cells.

### Knockdown of β-catenin induces cell cycle arrest in H295R cells via CDK inhibitors and MAPK pathway

**Figure 3:**
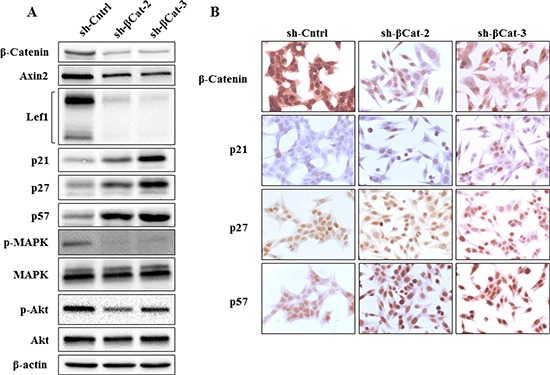
Expression of β-catenin, axin2, lef1, p21, p27, p57, p-MAPK and p-Akt in H295R cells treated with β-catenin shRNA **(A)** as detected by western blot analysis, shRNA targeting β-catenin in H295R cells down-regulated β-catenin as well as expression of axin2, lef1, p-MAPK and p-Akt, whereas p21, p27 and p57 expression were up-regulated. **(B)** immunohistochemical detection of β-catenin, p21, p27 and p57 expression in H295R cells treated either with sh-Cntrl, sh-βcat-2 or sh-βcat-3. β-Catenin expression decreased following treatment of H295R cells with both shRNA targeting β-catenin, whereas p21, p27 and p57 expression increased.

To further measure suppression of the Wnt/β-catenin pathway following shRNA treatment, the protein lysates were examined for the protein expression of axin2 and lef1. *Axin2* and *Lef1* are well-established direct downstream transcription targets of canonical Wnt/β-catenin pathway whose expression is induced by β-catenin [10, 11]. We found that shRNA directed against β-catenin strongly reduced expression of axin2 and lef1, demonstrating inhibition of β-catenin function (Figure 3A). Then, to understand the mechanisms by which β-catenin shRNA induces S and G_2_/M phases arrest, we studied the expression of p21^Cip1^ (p21) [12], p27^Kip1^ (p27) [13] and p57^Kip2^ (p57) [14] which belong to the Cip/Kip family of cycling dependent kinases inhibitors (CDKIs). They are important regulators of the cell cycle by binding and inhibiting several cyclin-dependent kinase/cyclin complexes [15] involved in the control of G_1_ phase, G_1_/S phase transition and M phase. As shown on Figure 3A and 3B, the protein expression of all three CKIs has substantially increased in sh-βcat-2 and -3 cells compared to the sh-Cntrl which is consistent with their role in restraining cell cycle progression.

MAP kinase pathway activation is known to regulate various cellular responses and, in particular, its role in G_1_/S and G_2_/M phases transition, the inhibition of CDKIs, and cell proliferation is well established [16–21]. We then analyzed whether p-MAPK expression level was affected in our cell populations. The knockdown of β-catenin expression induced almost complete p-MAPK inactivation in sh-βcat-2 and -3 cells compared to sh-Cntrl cells (Figure 3A).

**Figure 4:**
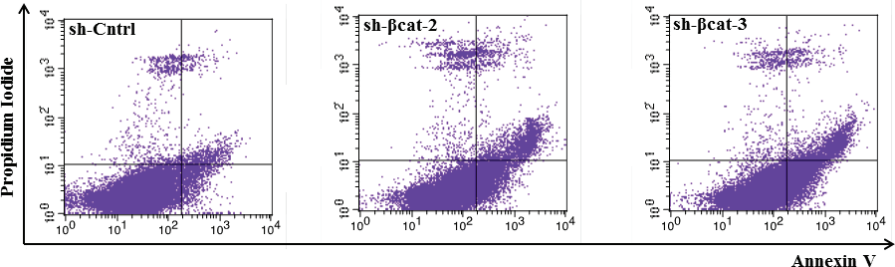
Loss of β-catenin is sufficient for increased apoptosis Representative FACS histograms indicating the percentage of apoptotic H295R-sh-Cntrl, -sh-βcat-2 and -sh-βcat-3 cells as determined by binding of FITC-conjugated Annexin V.

Akt is the primary mediator of PI3K signaling pathway and has a number of downstream substrates which explains its role in cell cycle progression and cell survival [22]. It also cooperates with p-MAPK to fulfil its function in regulating the G_1_- to S-phase transition. Phosphorylated Akt decreased in both sh-β-catenin cell lines compared to the control (Figure 3A). Furthermore, to investigate whether the decrease in p-Akt levels might induce apoptosis of sh-βcat cells, apoptotic death was assessed using flow cytometry analysis of annexin V stained cells. As shown in Figure 4, knockdown of β-catenin expression with shRNA plasmid promotes apoptosis in H295R cells. The apoptosis rates were 1.76 ± 0.51, 7.06 ± 1.46 and 7.01 ± 1.76% for sh-Cntrl, sh-βcat-2 and sh-βcat-3, respectively.

### β-catenin gene silencing induced changes in the morphology and in markers of epithelial and mesenchymal phenotypes of H295R cells

**Figure 5:**
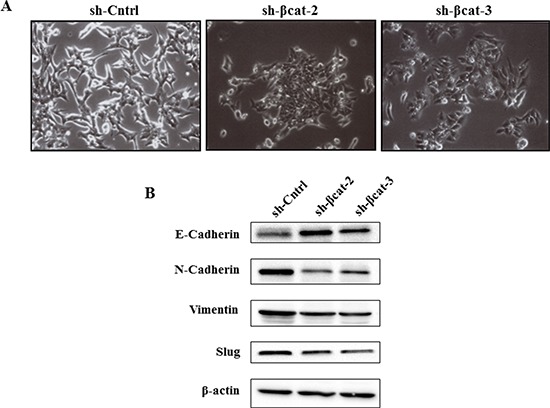
β-Catenin knockdown leads to changes on cellular morphology and the reversal of EMT of H295R cells **(A)** phase contrast images show the morphology of the indicated cell lines. **(B)** expression levels of E-cadherin, N-cadherin, vimentin, and slug in H295R-sh-Cntrl, sh-β-cat-2, and sh-βcat-3 cells, cells examined by immunoblotting. β-Actin expression is used as a loading control.

Cells expressing control shRNA grew in monolayer culture and displayed an elongated fibroblastic morphology resulting in reduced cell-cell contacts and cell scattering (Figure 5A). In contrast, cells depleted in β–catenin had a rounded shape, typical of an epithelial cobblestone appearance (Figure 5A) suggesting that these cells had potentially undergone a reversal EMT. To confirm whether, in addition to the observed morphologic changes, the molecular alterations associated with a reversal EMT occurred on loss of β-catenin, we assessed the expressions of epithelial and mesenchymal phenotype markers in sh-Cntrl, sh-βcat-2 and -3 cells. On shRNA-mediated loss of β-catenin, expression of epithelial protein E-cadherin was greatly increased while expression of mesenchymal proteins such as N-cadherin and vimentin was reduced (Figure 5B). Moreover, the expression of slug (snail2), a repressor of E-cadherin transcription and central mediator in EMT in multiple cancers, was lower in β-catenin knockdown cells compared to control cells. These observations indicate that loss of β-catenin protein in cells results in the acquisition of a differentiation state associated with passage through a reversal EMT.

### Functional differences in cell motility, and anchorage-independent growth

**Figure 6:**
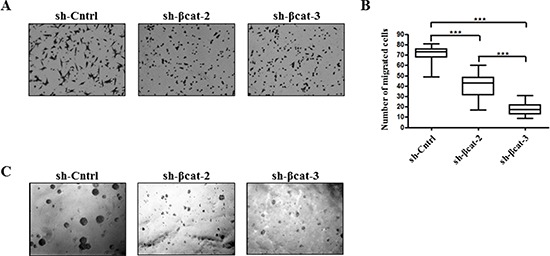
Loss of β-catenin leads to reduced migration and anchorage-independent growth **(A)** H295R-sh-Cntrl, -sh-βcat-2 and -sh-βcat-3 cells were induced to move through uncoated transwell membranes. After 15 h, the migrated cells were fixed, stained, and photographed. Representative photographs of Transwell membranes showing stained migrated cells from motility experiments. **(B)** quantification of motility experiments done by counting the number of migrated cells. Box-and-whisker plot of migrated H295R-sh-Cntrl, -sh-βcat-2 and -sh-βcat-3 cells.***P < 0.001, *n* = 2. **(C)** soft agar colony formation of H295R-sh-Cntrl, -sh-βcat-2 and -sh-βcat-3 cells. Figure shows representative photomicrographs of colonies generated by cells seeded at 1 × 10^3^ cells/ml in triplicate.

In order to determine whether the β-catenin expression level was functionally correlated with the *in vitro* capacity of cells to express properties ascribed to the acquisition of malignant phenotype, we carried out *in vitro* chemotaxis assays to characterize the control and β-catenin-depleted H295R cells. More specifically, we used modified Boyden chamber assays to evaluate the migratory ability of these cell populations. Whereas control cells displayed sustained migratory property, β-catenin loss resulted in a significant reduction in migration (Figure 6A and 6B). This finding suggests that the knockdown of β-catenin inhibited essential properties for the transformed cells to disseminate away from the primary tumor. Next, we assessed the ability of each of these cell lines to proliferate in an anchorage-independent fashion, a hallmark of *in vitro* transformation [23]. Both H295R β-catenin depleted cell populations generated only a few, very small abortive colonies. In contrast, sh-Cntrl cells formed large colonies in soft agar (Figure 6C). These results indicate that EMT reversal upon β-catenin depletion impedes the transformed phenotype of H295R adrenocortical carcinoma cells.

### Primary tumor formation of β-catenin-inhibited cancer cells

**Figure 7:**
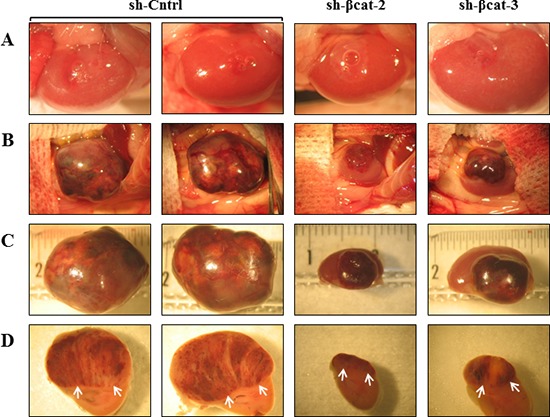
Loss of β-catenin leads to reduced malignant behavior of tumors formed after transplantation After growth in culture, 1×10^6^ of H295R-sh-Cntrl, -sh-βcat-2 and -sh-βcat-3 cells were implanted under the kidney capsule of scid mice. **(A)** macroscopic appearance of the mouse kidney immediately after transrenal injection of the cells (magnification x3.75). **(B)** macroscopic appearance of the same mouse kidney at 46 days. The tumor masses found to have resulted from growth of the transplanted cells were photographed. The transplantation of H295R-sh-Cntrl cells produced continuously expanding tissue masses surrounding the kidney which was no longer visible. The H295R-sh-βcat-2 and -sh-βcat-3 cells produced tumors on surface of mouse kidney (magnification x1.5). **(C)** The kidneys bearing tumors were retrieved and photographed on a ruler (magnification x2.4). **(D)** tumor and kidney were cut transversally showing internal tumor above the mouse kidney in axial view (arrows) (magnification x3).

**Figure 8:**
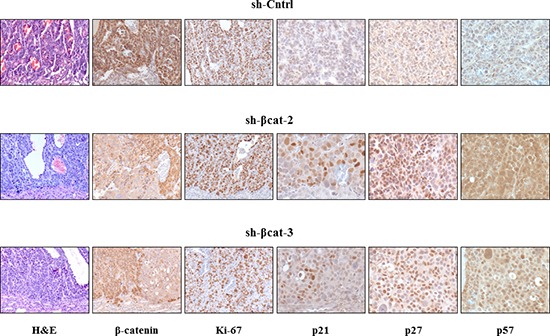
Histological appearance of tumors formed after transplantation of H295R-sh-Cntrl, -sh-βcat-2 and -sh-βcat-3 cells Paraffin-embedded tissues were sectioned, stained for hematoxylin and eosin (x100) and immunostained for β-catenin (x100); Ki-67 (x100); p21 (x200); p27 (x200); and p57 (x200).

**Figure 9:**
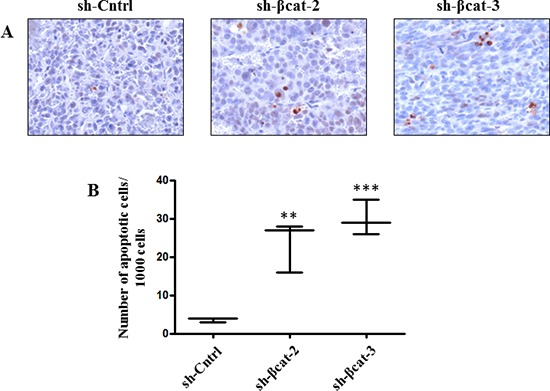
Cell death in tumor formed from H295R-sh-Cntrl, -sh-βcat-2 and -sh-βcat-3 **(A)** sections were used for cleaved caspase-3 detection of apoptotic cells. Magnification x200. **(B)** quantification of apoptosis experiments done by counting the number of cleaved caspase-3 positive cells. Box-and-whisker plot of apoptotic H295R-sh-Cntrl, -sh-βcat-2 and -sh-βcat-3 cells. **P < 0.01, ***P < 0.001, *n* = 3.

To determine whether β-catenin perturbation affects primary tumor growth, we transplanted sh-Cntrl cells and the two β-catenin-inhibited derivative cell populations beneath the kidney capsule of Scid mice (Figure 7A). The transplantation of sh-Cntrl cells resulted in highly neoplastic tumors with high tumor growth rates. Ultimately, by day 46, the tumor masses surrounded and invaded the kidney, destroying the organ (Figure 7B and 7D). At the time of sacrifice, tumors attained dimensions of 2 cm and weighed 1.8 to 2.5 g. In contrast, the tissues formed following the transplantation of sh-βcat-2 and -3 cells remained small and showed no invasive properties with respect to the kidney parenchyma (Figure 7B, and 7D). By 46 days, as shown in Figure 7C, tumors attained dimensions between 0.5 to 0.8 cm and weighed 0.6 to 0.9 g (kidney included). Histological examination of the control tumor demonstrated the invasive property of the cells as shown by identification of renal tubules engulfed into the tumor and by the detection of groups of tumor cells at remote distances from the primary tumor mass located between kidney tubules (Figure 8). In contrast, the histological appearance of the tumors formed from sh-βcat-2 and -3 cells displayed a clear boundary with the kidney parenchyma (Figure 8). We confirmed by immunohistological staining that β-catenin suppression was maintained at the time of sacrifice in tumors formed from sh-βcat-2 and -3 cells (Figure 8). Examination of Ki-67 expression in serial sections showed that the transplanted sh-Cntrl, sh-βcat-2 and -3 cells have an extremely high proliferation rate (Figure 8) which is in consonance with the *in vitro* findings (Figure 2C). These properties - retaining a small size despite high proliferation rates - suggested that there must also be an alteration in the regulation of cell growth through cell cycle control mechanisms and/or a high rate of cell death. Both were confirmed using immunohistochemistry. Whereas nuclei that showed a positive reaction for p21, p27 or p57 were uncommon in tissues formed from control cells, the expression of these CDK inhibitors was markedly up-regulated in tumors formed following sh-βcat-2 and -3 cells transplantation (Figure 8). Furthermore, to clarify whether β-catenin depletion might activate apoptosis as part of the mechanisms limiting tumor growth, we investigated the expression of cleaved caspase-3. Nuclei that showed a positive reaction by cleaved caspase-3 staining were present in tumors formed from sh-βcat-2 and -3 cells, but were uncommon in tumors formed from sh-Cntrl cells (Figure 9A). Moreover, there was no difference in cell apoptosis induced by either sh-βcat-2 or sh- βcat-3 (Figure 9B).

## DISCUSSION

Malignant adrenocortical tumor is a highly invasive and challenging tumor. Currently available therapies offer only limited benefits for patients with ACCs. There is an urgent need to develop new therapeutic approaches by better understanding the molecular pathogenesis of ACCs. The Wnt signaling pathway has crucial roles in the regulation of cellular, embryological and physiological activities [24]. A large majority of diverse cancer types express mutated pathway components, underscoring the importance of the Wnt signaling to tumorigenesis [25]. Accumulation of β-catenin protein has been identified as a key oncogenic process in the development of cancers. Ragazzon et al. [26] examined the expression of β-catenin in ACCs by immunohistochemistry and found that their expression levels were elevated with the ascending order of the tumor grade. Moreover, ACC patients with β-catenin cytoplasmic and nuclear accumulation had a poorer outcome than those with membranous β-catenin [5, 27]. Most of the *CTNBB1* gene mutations occur in exon 3, affecting the phosphorylation sites for GSK3β and then, preventing β-catenin proteasomal degradation. However, the frequency of missense mutation is significantly lower than the cytoplasmic and nuclear accumulation of β-catenin, which, taking into account these limited data, suggests that mutations of the *CTNBB1* gene may be not a major molecular event that results in the accumulation of β-catenin in the aberration of the Wnt/β-catenin pathway in ACCs [4, 5, 28].

As β-catenin accumulation is the hallmark of an aberrant activation of canonical Wnt pathway, the down regulation of β-catenin expression has attracted much attention as gene therapy target for carcinomas, including colon cancer [29] and multiple myeloma [30, 31]. In the current study, we determined how the long term silencing effect of β-catenin expression by RNA interference in established human adrenocortical cancer cells altered their growth both in culture and in Scid mice.

The cell cycle is tightly regulated through a complex network of positive and negative molecules such as cyclin-dependent kinases, cyclins and CDKis. The G_1_/S and G_2_/M phases transitions have been shown to be governed by activated CDK-cyclins complexes, whose activities can be suppressed by binding to CDKis such as p21, p27 and p57 [32, 33]. Although the loss of both G_1_/S and G_2_/M checkpoints are required for increased growth and proliferation in carcinomas, β-catenin loss induction of p21, p27 and p57 was associated with cell cycle arrest at the S and G_2_/M phases, accompanied by a decrease in the number of cells in G_1_. Interestingly, despite the increase expression of the CDKis, the number of cycling cells was not changed as evidenced by Ki-67 expression suggesting that the progression of the H295R cells through the cycle is hold up. Furthermore, our results are in contrast with the data reported by Gaujoux and colleagues in the same cell line [34] showing an accumulation of cells in G_1_ phase without a difference in proliferation. We do not know the reason for the discrepancy in the cell cycle phase arrest, however in both studies the tumor burden is reduced once the cells were transplanted in mice.

Knockdown of canonical Wnt/β-catenin simultaneously suppresses the activation of Ras/MAPK and PI3K/Akt pathways, which are two of the major downstream effectors of tyrosine kinase receptor such as EGFR, suggesting the cross-talk between these pathways. EGFR has been shown to be overexpressed in 76 to 100% of ACCs [35–37] however, mutations in *EGFR* or in downstream pathways are extremely rare [37, 38]. Pharmacological inhibition of EGFR with tyrosine kinase inhibitors has shown no improvement in the treatment of advanced ACCs [39]. One possible explanation for these disappointing data is that EGFR expression was truly causally involved in the generation of the malignant phenotype and that at the later stages of the disease progression, it might only be bystander and then, the targeted therapy may be inefficient. Moreover, ACCs typically display numerous genetic alterations that are likely to require impairment of multiple molecular pathways for successful treatment. Simultaneous targeting of multiple specific biochemical pathways may represent a decisive improvement in the therapeutic strategy of ACCs. Clearly, further studies are needed to explore the possible interaction of Wnt/β-catenin signaling and EGFR overexpression in ACCs.

While the process of EMT plays a pivotal role for the progress of embryonic development and later for wound healing, the abnormal activation of EMT programs can lead to tissue fibrosis and organ failure and, cancer progression [40–42]. Whereas significant progress has been made in identifying molecular alterations responsible for adrenocortical tumor growth, the change in expression of biomarkers that the cancer cells often acquire to disseminate from the primary tumor site to form metastasis has never been documented in ACCs. We observed that adrenocortical cancer cells may gain mesenchymal characteristics, associated with the expression of mesenchymal markers (e.g., N-cadherin and vimentin) as part of the process towards the acquisition of a fully transformed phenotype. Moreover, the progression of benign tumors to invasive metastatic cancer involves repression of epithelial marker E-cadherin through the expression of the transcription factor slug, leading to an impairment of its adhesive function [43]. Thus, the loss of E-cadherin expression along with the gain of N-cadherin and vimentin expressions trigger EMT in H295R cells. As EMT is a dynamic and reversible process which is complemented by mesenchymal-epithelial transition [44], we analyzed changes in expression of EMT regulators in our cell populations. Interestingly, knockdown expression of β-catenin in H295R cells leads to the reversal of EMT phenotype by up-regulation in the protein expression of E-cadherin and by down-regulation of N-cadherin, vimentin and slug. This change in cadherin expression is referred to as ‘cadherin switch’ and is increasingly used to monitor EMT [45]. These results strongly suggest the importance of β-catenin signaling in tumor cell aggressiveness through the acquisition of EMT phenotype in adrenocortical cancer cells. Therefore, targeting β-catenin signaling by novel approaches would be useful for reversing the EMT phenotype, which would likely result in the reversal of neoplasm recurrence and elimination of cancer cells.

Following transplantation, we found that β-catenin shRNA inhibits the tumor development and prevents invasion of H295R adrenal cancer cells xenografted in Scid mice. Our data suggest that shRNAs may have therapeutic potential for inhibiting the expression of genes that enhance the growth of tumors. The ability of shRNAs directed against β-catenin to result in prolonged suppression of β-catenin levels in cell culture and reduce *in vivo* growth of H295R cells (up to 46 days) suggests that their expression and their efficacy are stable over long periods of time. One of the current challenges for realizing the potential of RNA interference therapeutics in treating cancer is the development of successful delivery systems of small interfering RNA molecules into the tumors to induce target gene knockdown [30, 46–49].

In summary, results of the present study demonstrate that knockdown of the canonical Wnt pathway using β-catenin shRNA effectively suppresses the malignant adrenocortical tumor growth and may represent a potential therapeutic target for ACCs. Moreover, the H295R cell line might be useful in preclinical research for the optimization of rationally designed cancer therapy that targets several specific signaling pathways since the use of one drug may be ineffective to induce an objective response. Finally, interfering with Wnt signaling in ACC patients irrespective of the mutational status of β-catenin might be effective since decreased growth, survival and tumor volume were observed in hepatocellular carcinoma and myeloma cell lines expressing non mutated β-catenin [30, 50].

## MATERIALS AND METHODS

### Cell culture

Human H295R adrenal cancer cell line was maintained in DMEM/Ham's F-12 1:1 supplemented with 2% Serum replacement 3 (SR3, Sigma), 1% ITS (Becton Dickinson) and antibiotics, and grown at 37°C under an atmosphere of 5% CO_2_-95% air.

### Contructs, transfections and treatments

The β-catenin shRNAs (Sigma) were designed as a 57-mer containing a hairpin-loop and 21 nucleotides derived from the β–catenin gene (GenBank accession number NM_001904). Three target sequences were used to generate shRNA constructs against β–catenin which encode nucleotides 1302–1323 (AGGTGCTATCTGTCTGCTCTA), 1961–1982 (CGCA TGGAAGAAATAGTTGAA) and 2333–2351 (GCTTG GAATGAGACTGCTGAT). The double-stranded oligodeoxyribonucleotides were then denominated sh-βcat-1, sh-βcat-2 and sh-βcat-3, respectively. A non-target shRNA control plasmid (Sigma) was used as a negative control (sh-Cntrl). The targeting sequence of sh-Cntrl was CAACAAGATGAAGAGCACCAA and did not target any human gene but can activate RNA-induced silencing complex (RISC) and the RNAi pathway. Each shRNA was cloned into the pLKO.1-puro lentiviral vector. Lentiviruses were generated by transfection of 80% confluent HEK293T cells with MISSION^TM^ lentiviral packaging mix (Sigma) and the shRNA transfer plasmid using FuGENE^®^ 6 (Roche Diagnostics) as a transfection reagent in SR3-free media. At 16 h post-transfection, the media was changed for complete media. Lentiviruses were harvested at 24 and 48 hours and filtered. H295R cells were plated in 24-well plates for 24 h at 2 × 10^4^ cells/ml. The cells were then transduced with various volume of lentiviral preparation. After 24 h incubation, a stable pooled population of infected cells was selected with 1 μg/ml puromycin for 3 days, expanded and examined for stable β-catenin silencing.

### Western blot analysis

Cells treated with β-catenin or control shRNA were used for the preparation of total cell lysates. Cells were rinsed in PBS and lysed in RIPA buffer (10 mM Tris-HCl, pH 7.4, 150 mM NaCl, 1% TritonX-100, 0.5% deoxycholic acid, 0.1% SDS) supplemented with protease inhibitor cocktail (#P8340; Sigma) for 10 minutes on ice, scrapped from the culture dish, and cleared with centrifugation in a microfuge tube for 20 min at 4°C. Extracts were analyzed for protein concentration by Bradford assay. Equal amounts (20 μg) of total cell protein were separated by 15% SDS-PAGE gel and transferred to nitrocellulose membrane (Bio-Rad Laboratories). Filters were blocked for 1 hr at room temperature in 5% dry milk in Tris Buffered Saline (TBS), and subsequently incubated with primary antibodies as follows: anti-β-catenin (BD Biosciences) 1:1200, anti-axin2 (Abcam) 1:1000, anti-lef1 (Cell Signaling Technology), 1/1000, anti-p21 (EMD Millipore), 1:200, anti-p27 (Santa Cruz Biotechnology), 1:200, anti-p57 (Santa Cruz Biotechnology), 1:200, anti-p-MAPK (Promega), 1:1000, anti-MAPK (Sigma), 1:40000, anti-p-Akt (Cell Signaling Technology), 1:1000, anti-Akt (Cell Signaling Technology), 1:1000, anti-E-cadherin (BD Biosciences), 1:1000, N-cadherin (BD Biosciences), 1:1000, anti-slug (Cell Signaling Technology), 1:1000, anti-vimentin (Sigma), 1:200 and β-actin, 1:5000 in TBS containing either 0.1% Tween 20 or 0.1% Tween 20 and 5% BSA at 4°C overnight. After several washes, a secondary peroxidase conjugated antibody (Thermo Scientific) was used at a 1:10000 dilution. The membrane was washed in TBS-5% Tween 20 and the proteins were detected using an enhanced chemiluminescence detection system (Perkin Elmer).

### Cell growth assay

The growth rate of control and sh-treated H295R cells was evaluated by the measurement of cell number. H295R cells were plated in triplicate in 12-well plates at the initial cell density of 8 × 10^4^ cells/ml, and cultured in medium without SR3 for 24 h. After the medium was replaced, the cells were then incubated for 48 hours, trypsinized and the number of cells determined every 24 hours for up to 5 days on a hemocytometer.

### Cell migration assay

The Transwell chambers (Corning) with 8.0-μm pore polycarbonate membrane insert were used to assay migration activity of H295R cells. Each chamber was placed into a well of a 24-well plate. 2 × 10^5^ cells were suspended in 100 μl serum-free DMEM/F12 and placed into the upper compartment of the chambers. The well was filled with 600 μl DMEM/F12 supplemented with 5% SR3 as a chemo-attractant. After 15 h incubation at 37°C, 5% CO_2_, cells were removed from the upper surface of the polycarbonate membrane with a cotton swab. The invaded cells on the lower surface were fixed with 4% paraformaldehyde (PFA) and stained with 1% Crystal Violet for 20 min. After air-drying at room temperature, four randomly selected fields were photographed with an inverted phase contrast microscope (Zeiss) at x200 magnification and the migrated cells were counted on a computer screen. The mean cell number of four randomly selected fields was used for statistical analysis.

### Soft agar colony formation assay

Equal volumes of agarose (1.6%) and growth medium were mixed and plated to form a bottom layer (0.8% agar growth medium) in 6-well plates. Cells (1 × 10^3^ cells/ml) were suspended in regular medium, mixed with equal amount of 0.6% agarose and cell suspension-agar mix (2 ml) seeded as top layer in each well. Plates were incubated under normal culture conditions for 3 weeks for colony formation. Colonies were observed and photographed at x100 magnification in each plate.

### Assessment of cell-cycle and apoptosis analysis with flow cytometry

Cells from the three groups were harvested by trypsinisation and centrifugation. After counting, 1 × 10^6^ cells were washed twice in PBS-EDTA (2 mM), fixed in cold 70% ethanol for 30 min. The cells were washed twice in PBS-EDTA and resuspended in PBS containing 1 mg/ml propidium iodide (Sigma) and 10 mg/ml RNase A (Sigma) for 30 min at room temperature in the dark. The percentage of cell population in each phase of the cell cycle was measured using FACSCalibur (Becton Dickinson) and the results were analysed with the software CellQuest (Becton Dickinson).

The Annexin V-Cy5 Apoptosis detection kit (Abcam) was used to analyze quantitatively apoptosis of H295R cells. Cells were washed twice with cold PBS and resuspended in annexin-binding buffer. Annexin V-Cy5 and propidium iodide were added and the tubes were incubated at room temperature for 5 min in the dark and then analyzed by flow cytometry (Becton Dickinson).

### Cell transplantation in scid mice

Scid immunodeficient mice were purchased from Taconic. The animals were maintained in our animal facility, housed under controlled temperature and 12 h light-dark cycle conditions with regular unrestricted diet. All procedures were conducted according to the institutional guidelines and those formulated by the European Community for the Use of Experimental Animals. Under tribromoethanol anesthesia, 6 week-old mice were surgically operated [51, 52] and 1 × 10^6^ cells were transplanted under the renal capsule. 5 mice were used per cell population. Post-operative care for the animals consisted of the administration of analgesics and a mixture of antibiotics in the drinking water for 4 days [51]. Animals were killed 46 days following cell transplantation and subjected to necropsy.

### Histological and immunohistochemical analyses

The tumors were fixed in 4% PFA and embedded in paraffin. Microtome sections (5 μm thick) were stained with H&E for histological analysis. For IHC examinations, sections were deparaffinised, rehydrated and subjected to antigen retrieval in citrate buffer (10 mM, pH 6.0). The slides were incubated for 1 hour at room temperature with β–catenin, Ki-67, p21, p27 and p57 primary antibodies as described earlier and detected with a biotin-conjugated anti-mouse IgG or anti-rabbit IgG antibodies according to the primary antibody in use and an avidin- biotin-peroxidase complex (Vector Laboratories). Sections were lightly counterstained with hematoxylin. Apoptotic cells were detected on paraffin-embedded tumor sections by an anti-cleaved Caspase-3 rabbit monoclonal antibody (Cell Signaling Technology) at a dilution of 1:200.
